# The Cause of Divorce among Men and Women Referred to Marriage and Legal Office in Qazvin, Iran

**DOI:** 10.5539/gjhs.v4n5p184

**Published:** 2012-08-27

**Authors:** Ameneh Barikani, Sarichlow Mohamad Ebrahim, Mohammadi Navid

**Affiliations:** 1 Department of Community Medicine, Qazvin University of Medical Sciences, Tehran, Iran; 2 Department of Psychology, Qazvin University of Medical Science, Tehran, Iran; 3 Department of Community Medicine, Tehran University of Medical Sciences, Tehran, Iran

**Keywords:** divorce cause, marriage, Iran

## Abstract

**Background:**

Marital separation and divorce can be the most unpleasant event in the adult’s life, and families will be hurt by divorce event. The prevalence of divorce has been increased in last decades. Therefore, this study was conducted to identify the divorce cause among the divorce seeking men and women in Qazvin, Iran.

**Method:**

This cross-sectional study was conducted among 572 (400 women and 172 men) subjects who requested for divorce and were referred to divorce and marriage office of Qazvin province during 3 month in 2009. Data were collected by self – administered questionnaire, interviewing subjects and using Likert scale. Data were analyzed by Chi- Square test and Mann-Whitney (SPSS version 16).

**Results:**

The participants of the study included 400 women (26.5±7.4 years) and 172 men. In view points of women the primary wrong mate selection was main cause of divorce (59.8%), but the men believed that the families and relatives interference was the main reason for separation (43.7%). Among the respondents, mean score of “dependency to their families” and “unmet emotional needs” were 3.44±1.6 and 3.86±1.4 respectively. In addition mean score of infertility among men and women were 1.37±1.0 and 1.29±0.9 respectively.

**Conclusion:**

Wrong mate selection, unmet emotional needs, families’ interference, and “dependency to families” are more important factors than traditional factors which are sexual or physical factors.

## 1. Introduction

Family is the basic and essential unit of life that gives safety and comfort to their members. Marriage is necessary for forming of family. Marriage in Iran has several stages, including choosing spouse, offering marriage, engagement ceremony and wedding party. Normally both male and female choose his/her spouse by recommendation of his/her family. Two approaches for marriage are “arranged marriage” and “modern marriage”. In “arranged marriage”, the girl is introduced by male family or other relatives and then they begin their assessment and getting information about each others through neighbors, colleagues, relatives or friends to become familiar. In modern method the boys and girls meet each other in educational or work places and then the process will be with informing their families and consult with them. In Iranian marriage the issue of “Mahrieh” which is based on “Islamic law” is very important. This is the sum value of cash and jewelry which is payable to the bride (Aghajanian, 2001; Moghadam, 1994). The aim of engagement and the pre-marriage duration is that this new partner becomes more familiar with each other. The acceptable period for pre- formal marriage is 6-24 months.

In traditional families decision for marriage is made by parents. The partners’ opinion may not be important (Aghajanian, Tashakkori, & Mehryar, 1996), that causes primary wrong selection. In other words new couples that begins their new life does not have any idea about criteria and appearance of his/her spouse, which may leads to problem and divorce later in their life. There are several criteria for marriage that differs in cultural context. Mean age of marriage that is reported by statistical office is 26.1 and 22.6 years among boys and girls respectively (Iran Statistical Center, 2010). The mean ages of marriage have been increased in comparison to last decades. In Iran most of couples live independent, but in traditional families especially in rural area they may lives with their parents (Aghajanian, 2001).

Marital separation and divorce can be the most difficult event in an adult’s life. Divorce affects are economically, mentally, emotionally, physically and it exhibits depression and anxiety (Kurdeck, 1991; Amato, 1994). Several studies conducted during the past three decades have shown that children with divorced parents have an elevated risk of problems, such as physical disorders, emotional disturbances, difficulties with social relationships and academic failure (Aghajanian et al., 1996). Because of the political and policy implications of the economic situation associated with divorce, much more attention has focused on its economic impact. In the USA, Canada, and most other countries, women, following divorce generally experience a decline in their economic situation (Amato, 1994; Braver & Connel, 1988; Burkhauser, Dunlan, & Houser, 1991). Because of low economic activity and social status of Iranian women and their dependency to the men for living cost and social protection, divorce carries particularly heavy costs and consequences for Iranian women.

In the Islamic laws, in case of divorce, the men have the right to custody the children of age 3 years and older (in case of boys) and 7 years and older (in case of girl) (Aghajanian, 1986). Although not prohibited, divorce is strongly discouraged in Islam and not acceptable by Iranian culture, however, in recent years, divorce rate in Iran has been increased. Mean rate of divorce in Iran is 10.4 out of 100 marriages (Max 27), which is comparable with 25 divorce out of 100 marriage in the Paris (Hourriez, 2005). Tehran, the capital of Iran, had the highest rate of divorce in 2007 and 2009 with 22% and 27% respectively, and all of provinces experienced an increased rate over the period as well (KeshavarzHadad, 2010). More than 50% of divorces in Iran were in couples that their age difference was 0-5 years (Iran Statistical Center, 2010). During years 2004-2010 most of divorces were in ages of 25-29 years and 20-24years among male and female respectively. In Qazvin a province of Iran, during years 2006-2007 marriage and divorce rates were 5.5% and 4.2% respectively (Iran Statistical Center, 2010). Misunderstanding of each others, less attention to psychological needs, violence and sexual problems in a study in Tehran, were the most important reasons for divorce (Khalili, 2011). In addition, cultural difference, suspicious, drug abuse, insufficient understanding of each others, second marriage, interference of spouse family, age difference, loveless marriage, reduced sexual desire and sexual disease were important and could lead to divorce (Aghajanian, 1986). Iranian society has not any changes in civil procedures deal with family law during the past decades, but the attitudes of people towards divorce and social norms have tangibly changed, and it is significantly different across the provinces (KeshavarzHadad, 2010). Therefore, this study aimed to identify the divorce cause among the divorce seeking men and women in Qazvin, Iran.

## 2. Method & Material

This was a cross- sectional study and subjects were recruited from population of women and men who requested for divorce and were referred to divorce and marriage office of Qazvin province during 3 month (Aug.-Nov.) in year 2009. Before starting the main study, researchers conducted a pilot study with 20 subjects to ensure the validity of questionnaires. The pilot study of subjects later on included in total population of the study. All subjects who either requested or involved in divorce process with a total of 572 (400 women and 172 men) participated in this study. Data were collected using a self – administered questionnaire, which was supervised by a trained psychologist and were interviewed in consultation room of divorce and marriage office of Qazvin city. This questionnaire, consisted of two sections: first demographic questions such as: sex, job, education, duration of marriage, age difference with spouse, age of marriage, children numbers, income, place of life, relation with spouse, period of knowing each other before marriage, engagement duration, and how meet the spouse. Second section of questions was about the reason of divorce. The Likert scale (never=1, very low=2, low=3, high=4. very high=5) was administrated in this study. Likert scale was used to obtain the frequency of responses to causes of divorce and also to measure mean scores of “causes of divorce”. The Likert scale was checked by subjects for each question, on a 5-point scale, from +1 (“never”) to +5 (“very high”). The validity of questionnaire was confirmed by psychologist and community medicine specialist. Internal consistency was confirmed by Kronbach’s Alpha (0.92).

## 3. Statistical Analysis

Descriptive analyses were used to describe the participants’ details. Data were expressed as M±D and frequency of responses were determined using percentage. The analysis of data carried out by Chi-Square test and Mann-Whitney (SPSS version 16) for comparing of proportions and means with considering P<0.05.

## 4. Results

In this study 400 women and 172 men participated. Mean age of women was 26.5±7.4 and 83.3% of women were house keeper, meanwhile 8.1% of men were unemployed. Level of education in 90% of women and 82% of men was primary school or incomplete high school level (Table 1). In relation to assets, 31.4% men and 19.8% of women had private house. About 24.3% of them married with their relatives; but 63.3% women and 41.9% of men did not have any familiarity toward their spouse before marriage, however, 22.7% of men and 15.8% of women had 2 years familiarity before marriage. About 60% of women expressed that the “primary wrong selection” is main factor in divorce and considered =4 (“very high”) point as cause of divorce in Likert scale (Table 2), and 43.7% of men responded that the “effect of family interference” is main cause of divorce and marked +4 (“very high”) point in Likert scale (Table 3). Mean scores of “dependency to his or her family” and “unmet emotional needs” (feeling less) variables were higher than other factors among men and women (3.44±1.60, and 3.86±1.40 respectively). Also mean score of “infertility” as cause of divorce in both men and women were lower than other factors (1.37±1.00, and 0.90±0.30 respectively) (Table 4). However, Table 5 shows that “Primary wrong selection of spouse” and “dependency to his or her family” factors were the most important cause of divorce in view points of women and men respectively.

**Table 1:** Demographic characteristics of participants (N=567)

	Men	Women	P
Job			0.001
Unemployed or housekeeper	2.5(14)	59.9(332)	
Employed	26.4(148)	11.9(67)	

Age			0.001
=<30	17.5(100)	54.9(314)	
>30	12.6(72)	15(86)	

Engagement duration(months)			0.2
No	4.2(24)	6.8(39)	
<6	12.5(71)	28.2(161)	
>6	13.3(76)	34.9(199)	

Marriage duration(years)			0.6
<5	18(100)	40.6(226)	
5-10	6.7(37)	14.2(79)	
>10	5.4(30)	15.1(84)	

Age difference(years)			0.7
<2	7.2(40)	16.4(91)	
2-6	13.8(77)	30.6(170)	
>6	8.8(49)	23.2(129)	

Education			0.09
Low	6.5(37)	17.3(99)	
Middle	18.9(108)	45.9(262)	
High	4.7(27)	6.7(38)	

Age of Marriage(years)			0.001
<25	20.7(113)	64.1(350)	
25-30	6.6(36)	4.9(27)	
>30	1.8(10)	1.8(10)	

Meet duration before marriage(months)			0.001
No	12.7(72)	44.6(253)	
<6	7.4(42)	11.8(67)	
>6	9.3(53)	14.1(80)	

**Table 2:** Frequency and distribution of women response to cause of divorce (N=400)

	Very Low% (N)	Low% (N)	Middle% (N)	High% (N)	Very High% (N)
Unemployment of Spouse	34.2(137)	10.8(43)	8.5(34)	6.8(27)	39.8(159)
Economic problems	32(128)	10(40)	12.5(50)	10.5(42)	34(140)
Family interference	31.5(126)	4.5(18)	14.8(59)	13(52)	36.2(145)
Husband drug Abuse	52.8(211)	2.8(11)	5.8(23)	5.8(23)	33(132)
Husband alcohol use	62.8(251)	6.8(27)	12.2(49)	5.8(23)	12.5(50)
Family dependency	28.8(115)	8(32)	13.5(54)	12.8(51)	37(148)
dependency	30.2(121)	5.5(22)	10.8(43)	15.2(61)	38.2(153)
Un-responsibility	16(64)	5.8(23)	9.8(39)	15.8(63)	52.8(211)
Primary wrong selection	9.2(37)	5(20)	10(40)	16(64)	59.8(239)
Cultural difference	28.5(114)	7(28)	16.2(65)	16.8(68)	31.5(126)
Religious believes difference	36(144)	12(48)	13.2(53)	15.2(61)	23.5(94)
Unmet emotional needs(Feeling less)	12(48)	7(28)	13.5(54)	18(72)	49.5(198)
Arranged marriage	59.8(239)	5.8(23)	11.2(45)	6(24)	17.2(69)
Loveless marriage	41.2(165)	7(28)	14.8(59)	9(36)	28(112)
Adultery	68.2(273)	2.5(10)	4.5(18)	6.2(25)	18.3(73)
Second marriage	82.5(330)	1.5(6)	3.8(15)	2.8(11)	9.5(38)
Illiteracy of husband	61.2(245)	10.8(43)	13.2(53)	6(24)	8.8(35)
Bankrupt	77.5(310)	5(20)	6.2(25)	4(16)	7.2(29)
Criminal or smuggling behavior of husband	69.2(277)	3.2(13)	5(20)	6.5(26)	15.8(63)
Impotency	76(304)	4.2(17)	8(32)	5.2(21)	6.3(25)
Other sexual problems	78.2(313)	3.5(14)	7(28)	5.8(23)	5.5(22)
Psychological problem	34.8(139)	8.5(34)	12(48)	16.5(66)	27.8(111)
Suspicious	44(176)	4.2(17)	6.8(27)	13.2(53)	31.8(127)
Age difference	60(240)	11.8(47)	11.8(47)	7(28)	9.5(38)
Not paying living cost	31(124)	5.8(23)	9.8(39)	12.8(51)	40.8(163)
Not paying milk money(Mahrieh)	59.2(237)	4.2(17)	4.2(17)	4.8(19)	27.5(110)
Anger of husband	18.5(74)	6(24)	11.8(47)	17.8(71)	46(184)
Hitting	34(136)	4.8(19)	10(40)	11.2(45)	40(160)
Not paying attention to children	55(220)	4.2(17)	8.2(33)	11.8(47)	20.8(83)
Being stingy	42(168)	7.8(31)	14.5(58)	9(36)	26.8(107)
Unethical behavior	22.8(91)	7.8(31)	11.8(47)	17.5(70)	40.2(161)
Bulling	23.8(95)	5.8(23)	11(44)	17(68)	42.5(170)
Loss of sense	20.8(83)	4.5(19)	12.2(49)	17.5(70)	44.8(179)
Infertility	90(360)	1(4)	3(12)	1.5(6)	4.5(18)

**Table 3:** Frequency and distribution of men responds to cause of divorce (N=172)

	Very Low% (N)	Low% (N)	Middle% (N)	High% (N)	Very High% (N)
Family interference	30.2(52)	3.5(6)	12.2(21)	12.2(21)	41.3(71)
Family dependency	23.8(41)	7.6(13)	10.5(18)	16.9(29)	41.3(71)
dependency	39(67)	11(19)	12.8(22)	16.9(29)	20.3(35)
Un-responsibility	25.6(44)	5.8(10)	18(31)	22.7(39)	27.9(48)
Primary wrong selection	26.7(46)	4.7(8)	14.5(25)	19.2(33)	34.9(60)
Cultural difference	34.9(60)	9.9(17)	18.6(32)	9.9(17)	26.7(46)
Religious believes difference	48.3(83)	10.5(18)	15.7(27)	12.2(21)	13.4(23)
Unmet emotional needs(Feeling less)	18(31)	10.5(18)	16.3(28)	21.5(37)	32.6(56)
Arranged marriage	63.4(109)	10.5(18)	11(19)	5.2(9)	9.9(17)
Loveless marriage	50(86)	5.2(9)	8.7(15)	12.8(22)	23.3(40)
Adultery	77.9(134)	1.2(2)	4.7(8)	3.5(6)	12.8(22)
Illiteracy of wife	62.2(107)	16.9(29)	11(19)	5.8(10)	4.1(7)
Criminal behavior of wife	81.4(140)	6.4(11)	2.9(5)	3.5(6)	5.8(10)
Loss of sexual desire of wife	51.7(89)	5.2(9)	12.2(21)	11(19)	19.8(34)
Other sexual problems of wife	57.6(99)	10.5(18)	15.1(26)	11.6(20)	5.2(9)
Psychological problem	47.7(82)	5.2(9)	11.6(20)	20.9(36)	14.5(25)
Suspicious	41.3(71)	6.4(11)	14.5(25)	13.4(23)	24.4(42)
Age difference	53.5(99)	11(19)	18.6(32)	8.7(15)	8.1(14)
Wife’s anger	29.1(50)	11(19)	15.1(26)	19.8(34)	25(43)
Not paying attention to children	60.5(1.4)	5.8(10)	12.8(22)	12.2(21)	8.7(15)
Unethical behavior	26.7(46)	12.8(22)	10.5(18)	24.4(42)	25.6(44)
Addiction of wife	84.3(145)	1.2(2)	3.5(6)	4.1(7)	7(12)
Loss of sense	30.2(52)	16.3(28)	9.3(10)	19.2(33)	25(43)
Infertility	85.5(147)	2.9(5)	5.2(9)	1.7(3)	4.7(8)

**Table 4:** The mean score of common cause of divorce between men and women

College	Men		Women		P

	Mean	SD	Mean	SD	
Dependence to his or her family	3.44	1.6	3.21	1.6	0.1
Unmet emotional needs (Feeling less)	3.40	1.4	3.86	1.4	0.001
Primary wrong selection of spouse	3.30	1.7	3.18	1.6	0.4
interference of spouse family	3.30	1.7	3.18	1.6	0.4
Un-responsibility	3.21	1.5	3.83	1.5	0.001
Unethical behavior	3.09	1.5	3.43	1.6	0.02
Fiery temper(anger)	3.00	1.5	3.66	1.5	0.001
Loss of sense	2.92	1.6	3.60	1.5	0.001
Suspicious	2.73	1.6	2.84	1.7	0.4
Dependency	2.88	1.6	3.25	1.6	0.001
Cultural difference	2.83	1.6	3.15	1.6	0.03
Loveless marriage	2.54	1.7	2.75	1.6	0.1
Psychological problem	2.49	1.5	2.93	1.6	0.003
Loss of sexual desire	2.41	1.6	1.61	1.2	0.001
Other sexual problems	1.96	1.2	1.56	1.1	0.001
Religious believes difference	2.31	1.4	2.78	1.6	0.01
Not paying attention to children	2.02	1.4	2.93	1.6	0.01
Age difference	2.06	1.3	1.94	1.3	0.3
Adultery	1.72	1.4	2.03	1.6	0.02
Arranged marriage	1.87	1.3	2.15	1.5	0.04
Addiction of spouse	1.48	1.1	2.63	1.8	0.001
Illiteracy of spouse	1.72	1.1	1.90	1.3	0.1
Criminal or smuggling behavior	1.45	1.1	1.95	1.5	0.001
Infertility	1.37	1.0	1.29	0.9	0.3

**Table 5:** Ranking of the most important 10 cause of divorce in men and women

College	Men	Women
Dependence to his or her family	1	8
Unmet emotional needs(Feeling less)	2	2
Primary wrong selection of spouse	3	1
interference of spouse family	4	9
Un-responsibility	5	3
Unethical behavior	6	6
Fiery temper(anger)	7	4
Loss of sense	8	5
Suspicious	9	-
dependency	10	7
Cultural difference	-	10

## 5. Discussion

The aim of the current study was to identify the divorce cause among the divorce seeking men and women in Qazvin, Iran. The current study demonstrated that primary wrong selection of husband and high dependency of wife to her family in women and men were the most important factor of divorce respectively. Primary wrong selection refers to not having any idea about each others’ (couples) characteristics before marriage, that consequently may lead to different behavior in couples and divorce. Primary wrong selection, was the most important cause of divorce in Iran (Ghotbi, Holakoii Naini, Jazayeri, & Rahimi, 2004). In a traditional community, primary wrong selection was higher than modern community, because of less enough familiarity of boys and girls before marriage. In the past, these kinds of marriage may be last for long time of life, but today following greatly changes during last two decades toward modernization, the community values in Iran has been changed. In the traditional cultures man is dominant in the families, but in modern culture, women believed that, they are equal to the men in management of family and do not accept dominant of man. Distribution of power theory, believes that, man and woman for taking power have challenges, that could be lead to divorce (William, 1979). Women’s demand for their rights and economical independency, request of equality of power in family in front of un-acceptance of these situations by men, could lead to divorce.

Unmet emotional needs or feeling less, were the second important divorce cause in women and men. Each of men and women has own expectations toward his/her spouse, that if have not been met, make un-satisfaction. In Iran individuals do not like to show their true feeling and usually censored it. Fiery temper and interference of spouse family were the fourth important divorce cause, that is compatible with the behavioral theory. ^16^ Families interference was the second divorce factor in a study in Iran (Khajastehmehr, & Takrimi, 2009). When men and women begin their shared life, they would have new responsibility toward their spouse. Often families for supporting of their children, interference in their life, that not only causes problem, but also faster’s trend of divorce.

Sexual problems especially, low sexual desire in women, was one of important cause of divorce. Studies in Iran showed that sexual problems especially low sexual desire was one of the most important cause of divorce (Khalili, 2011; Karne, & Bradbury, 1995). In Iranian community men have liberty for sexual relation in out of marriage limitation that named temporary marriage (sigheh) (Haeri, 1994). This form of marriage that has not any limitation of permanent marriage, is accepted in view of religious and law. In this type of marriage, man and woman often agree privately and verbally to marry each other for a limited period of time, however it is not acceptable in the community. In our study, cultural difference, psychiatric disease, low sexual desire, adultery, addiction and criminal or smuggling behavior were significantly different between men and women.

The current study showed that the most of participants were adolescence. In other words, they married below 20 and 25 in women and men respectively, therefore they could ignore more responsibility that must accompanied with marriage. Reports of statistical office in Iran also showed that, most of divorce, had taken below age 20 and 25 years in women and men respectively. In majority of Arab countries, early and teenage marriage below 20 is above 50% (El-saadani, 2006). Numerous studies indicated that, the younger the age of marriage, the higher the probability that a marriage terminate in separation or divorce (Cherlin, 1977). Marriage in younger ages and not having any familiarity before marriage, could aggravated problems between couples. If men and women to get marry in younger ages, they have not obtained and have not learned life skills and responsibilities in life, therefore most of divorce had taken in the first years of marriage (Cherlin, 1977). Although the mean age of marriage in Iran had increased, but couples have not acquired skills of life before marriage.

Level of education in 90% and 82% of women and men were primary school and high school level. Contrary to the common believes, the prevalence of divorce was less in high educated people compared to low educated couples. Similar results have been documented in other studies (Lyngstad, 2004). In our study, participants with high educational level, showed higher score of interference of spouse family, dependency, cultural difference and sexual problems compared with lower educational level couples. Individuals with higher education, tend to have more independency in their life, and don’t accept interference of families or other relatives. In view of women, sexual problems, age difference, and infertility were not main cause of divorce. In view point of men infertility was not important factor for divorce. Our finding was opposite to other Islamic countries that infertility is considered a legitimate reason for divorce especially if the woman is infertile (El-saadani, 2006).

Our study suffered from limitations. For privacy reasons we couldn’t conduct the sampling in wider scale and we can not generalize the finding to whole population of the area. The study was conducted in a small city 120 km far away from capital with middle class culture and we can not generalize the finding to the couples living in either major cities or remote areas like villages. For dignity, sensitivity of issue and privacy reasons participants may not answered correctly and hiding information which may cause bias in data collection and results. Low educational level or illiteracy of some subjects was another problem during interview or filling questionnaire.

## 6. Conclusion

The presented study revealed that personal characteristics and socio-cultural factors such as wrong selection, unmet emotional needs, interference of families, high connection to families are more important than traditional factors (sexual or physical). This study has one important message for policy makers in divorce and families that for consistency of life, couples need to realize each other in all aspect of life, and that the authorities must create such environment.
